# Bone Plasticity in Response to Exercise Is Sex-Dependent in Rats

**DOI:** 10.1371/journal.pone.0064725

**Published:** 2013-05-31

**Authors:** Wagner S. Vicente, Luciene M. dos Reis, Rafael G. Graciolli, Fabiana G. Graciolli, Wagner V. Dominguez, Charles C. Wang, Tatiana L. Fonseca, Ana P. Velosa, Hamilton Roschel, Walcy R. Teodoro, Bruno Gualano, Vanda Jorgetti

**Affiliations:** 1 Nephrology Division, Medical School, University of São Paulo, São Paulo, Brazil; 2 Department of Sports, School of Physical Education and Sport, University of Sao Paulo, Sao Paulo, Brazil; 3 Department of Anatomy, Institute of Biomedical Sciences, University of Sao Paulo, São Paulo, Brazil; 4 Rheumatology Division, Medical School, University of São Paulo, São Paulo, Brazil; 5 Department of Physiological Sciences, Federal University of São Carlos, São Paulo, Brazil; Montana State University, United States of America

## Abstract

**Purpose:**

To characterize the potential sexual dimorphism of bone in response to exercise.

**Methods:**

Young male and female Wistar rats were either submitted to 12 weeks of exercise or remained sedentary. The training load was adjusted at the mid-trial (week 6) by the maximal speed test. A mechanical test was performed to measure the maximal force, resilience, stiffness, and fracture load. The bone structure, formation, and resorption were obtained by histomorphometric analyses. Type I collagen (COL I) mRNA expression and tartrate-resistant acid phosphatase (TRAP) mRNA expression were evaluated by quantitative real-time PCR (qPCR).

**Results:**

The male and female trained rats significantly improved their maximum speed during the maximal exercise test (main effect of training; p<0.0001). The male rats were significantly heavier than the females, irrespective of training (main effect of sex; p<0.0001). Similarly, both the weight and length of the femur were greater for the male rats when compared with the females (main effect of sex; p<0.0001 and p<0.0001, respectively). The trabecular volume was positively affected by exercise in male and female rats (main effect of training; p = 0.001), whereas the trabecular thickness, resilience, mineral apposition rate, and bone formation rate increased only in the trained males (within-sex comparison; p<0.05 for all parameters), demonstrating the sexual dimorphism in response to exercise. Accordingly, the number of osteocytes increased significantly only in the trained males (within-sex comparison; p<0.05). Pearson’s correlation analyses revealed that the COL I mRNA expression and TRAP mRNA expression were positively and negatively, respectively, related to the parameters of bone remodeling obtained from the histomorphometric analysis (r = 0.59 to 0.85; p<0.05).

**Conclusion:**

Exercise yielded differential adaptations with respect to bone structure, biomechanical proprieties, and molecular signaling in male and female rats.

## Introduction

The notion that “senile osteoporosis is a pediatric disease” has been increasingly accepted [1], [2], [3]. This suggestion relies on the observation that physical inactivity during growth has been related to an increased prevalence of osteoporosis-related fractures [2], [4], [5], [6]. In fact, mechanical strain exerts a pivotal role in skeletal growth and modeling. Both *in vitro* and *in vivo* studies have demonstrated that mechanical strain modifies intracellular bone signaling, partially by enhancing the expression of growth factors [7]. The mechanical stimuli-induced intracellular signaling has been suggested to potentially favor bone proliferation and the formation of bone matrix [7], although the actual mechanisms underlying these responses remain to be fully elucidated.

Exercise has been recognized as one of the most effective strategies to counteract low bone mass. Nonetheless, preliminary evidence suggests that the bone response to exercise may be sex-dependent. Differential bone plasticity in males and females may be rooted in the biologic properties of bone [1], [8]. In support of this concept, there is evidence suggesting sex-specific differences in the number of osteoprogenitor cells, hormone responses, and hormone regulation [9], [10], [11], which could potentially influence the normal growth of bone and its response to a given stimulus (e.g., dietary intervention, pharmacological treatment and physical activity). In humans, sex differences in bone size and strength have been suggested to be established in puberty as a result of the greater endocortical and periosteal expansion during the pre-pubertal years and the minimal endocortical contraction in males compared with the high endocortical contraction and inhibition of periosteal apposition in females after the pubertal growth spurt [1], [12], [13], [14]. As a consequence, volumetric density remains constant during growth and similar in both sexes, whereas the bone mass content (BMC) is approximately 20% higher in males than in females at the end of puberty as their bones are larger [1]. Thus, sex-related differences in bone strength are the result of the differences in shape and geometry [1], [15]. Recently, rodent studies have provided a growing body of evidence indicating that sex hormones and their receptors have an impact on the mechanical sensitivity of the growing skeleton. According to Callewaert et al. [8], androgen receptor and estrogen receptor-β signaling may attenuate the osteogenic response to mechanical strain in males and females, respectively, whilst estrogen receptor-α may stimulate the response of bone to mechanical strain in the female skeleton. Altogether, these findings support the hypothesis that skeletal sexual dimorphism in response to exercise may exist.

Thus, the objective of this study was to assess the potential sex-based differential bone plasticity in response to exercise in young rats.

## Materials and Methods

### Ethics Statement

All of the experimental procedures were approved by the local Research Ethics Committee (CAPPesq, permit number 806/05) and developed in strict conformity with our institutional guidelines and with international standards for the manipulation and care of laboratory animals. All of the surgeries were performed under sodium pentobarbital anesthesia, and all efforts were made to minimize suffering.

### Experimental Protocol

Male and female 12-week-old Wistar rats, obtained from our local breeding colony with initial weights ranging from 200 to 300 g, were used in this study. The animals were housed in individual cages in a light-controlled environment (12/12-h light/dark cycle) at a constant temperature (22°C) and humidity (25%), with free access to standard laboratory chow (Nuvital Nutrientes S/A, Curitiba, Brazil). After 7 days of acclimation, the animals were randomly divided into four groups as follows: *i)* female sedentary, *ii)* female trained, *iii)* male sedentary, and *iv)* male trained.

The animals were trained by using a treadmill training protocol adapted from Brooks et al. [16] and Ferreira et al. [17]. At the beginning of the training, all of the animals were submitted to a maximal speed test [16], [17]. The test was performed on a treadmill (ESD model 01; Funbec, São Paulo, Brazil) at an initial rate of 6 m/min with no slope. The rate was increased by 3 m/min every 3 min until exhaustion. The animals were judged to be exhausted when they could no longer maintain an upright position in the treadmill or continue running at the required pace. The training protocol intensity was set at 60% of the maximal velocity achieved in the test and was performed 5 times a week for 12 weeks. A mid-trial (i.e., week 6) maximal test was performed to adjust the training load. At the end of 12 weeks, all of the animals were submitted to a final maximal test and then anesthetized and euthanized through aortic exsanguination. The sedentary animals remained cage-confined without any physical training program throughout the protocol.

### Biomechanical Characterization

The right femur of each rat was removed, dissected free from the soft tissue and placed in saline 9% to - 80°C until mechanical testing. 12 hours before the test, frozen bones were thawed at room temperature and kept in saline. They were tested mechanically through a three-point bending protocol using an Instron Universal Testing machine, model 4444 (Instron Corp., Canton, MA, USA). The span of the supports was 21.7 mm, and the deformation rate was 0.5 cm/min. The load-deformation curve was analyzed, and the maximal force, resilience, stiffness and fracture load were calculated from the plot using specific software [18], [19].

### Bone Histomorphometry

A fluorochrome bone marker (i.e., oxytetracycline) was injected (25 mg/kg ip) on days 12 and 11 and on days 5 and 4 prior to the euthanization of the animals.

The length of the left femur (free from soft tissue) was measured using a caliper, weighed, immersed in 70% ethanol, and processed as previously described [20]. Using a Polycut S equipped with a tungsten carbide knife (Leica, Heidelberg, Germany), the undecalcified distal femurs were cut into sections of 5 µm and 10 µm in thickness. The 5-µm sections were stained with 0.1% toluidine blue, pH 6.4, and coverslips with mounting medium Entellan® (Merck, Darmstadt, Germany) and at least two nonconsecutive sections were examined for each sample. Static, structural and dynamic parameters of bone formation and resorption were measured at the distal metaphyses (magnification, ×250), 195 µm from the epiphyseal growth plate, in a total of 30 fields using a semi-automatic image analyzer and the software Osteomeasure (Osteometrics, Inc., Atlanta, GA, USA) specific for bone histomorphometry. The static and structural histomorphometric indices included the ratio of trabecular bone volume to total bone volume (BV/TV), the ratio of osteoid volume to total bone volume (OV/BV), and the osteoid thickness (O.Th). The percentage of the total trabecular surface was used to express the areas of eroded surface (ES/BS) and osteoid surface (OS/BS). We also determined the number of osteoblasts (N.Ob/T.Ar) and the number of osteoclasts (N.Oc/T.Ar) per tissue area, the number of osteocyte-occupied lacunae (stained) per bone area (N.Ot/B.Ar), the trabecular thickness (Tb.Th), the trabecular separation (Tb.Sp), and the trabecular number (Tb.N). Moreover, the mineral apposition rate (MAR) was determined by calculating the distance between the two oxytetracycline labels divided by the time interval between the two oxytetracycline administrations. The mineralizing surface (MS/BS), which is the rate of cancellous surface that has been mineralized, was calculated as the double-labeled surface plus one-half of the single-labeled surface. The evaluation of the rate of the total trabecular surface that was double-labeled and the bone formation rate (BFR/BS) completed the dynamic evaluation. All of the data were obtained in a blinded fashion. The histomorphometric indices are reported using the nomenclature recommended by the American Society for Bone and Mineral Research [21].

### Real-time PCR (qPCR)

The right tibias of each rat were removed, dissected, and crushed in a steel mortar and pestle set (Fischer Scientific International, Inc., Hampton, NH) that was pre-cooled in dry ice. Total RNA was extracted using Trizol (Invitrogen, Calbard, CA, USA), following the manufacturer’s instructions. Total RNA was reverse transcribed using RevertAid-H-Minus M-MuLV Reverse Transcriptase (Fermentas, Hanover, MD, USA) to synthesize the first strand cDNA, which was used as a template. The mRNA expression of proteins was determined by qPCR using the ABI Prism 7500 sequence detector (Applied Biosystems), as previously described [22]. The selected genes were Type I collagen (COL I) and tartrate-resistant acid phosphatase (TRAP). COL I was selected as it is a protein produced by the bone-forming cells, osteoblasts; whereas TRAP is an enzyme produced by the bone-resorbing cells, osteoclasts. The analysis was performed using a total volume of 20 µl containing the template (5 ng for Type I collagen and 10 ng for TRAP) and primers (450 ng). The primers used in this study were designed using the Primer Express software (Applied Biosystems) (Table 1) and synthesized (Integrated DNA Technologies, Coralville, IA, USA) specifically for qPCR. All of the Ct values were normalized using an internal control (β-actin mRNA). The relative gene expression quantification was assessed by the ΔΔCt method, as previously described [23]. The final values for the samples are reported as fold-induction relative to the expression of the control, with the mean control value being arbitrarily set to 1.

**Table 1:** Description and sequences of the genes selected for the study.

Gene	NM	Forward (F)	Reverse (R)
Type I collagen	053304	GCGAAGGCAACAGTCGAT	CTTGGTGGTTTTGTATTCGATGAC
TRAP	019144	GTTCCAGGAGACCTTTGAGGA	TCCAGCCAGCACGTACCA
β-actin	031144	AAGATTTGGCACCACACTTTCTACA	CGGTGAGCAGCACAGGGT

NM: NCBI accession number; TRAP: tartrate-resistant acid phosphatase.

### Statistical Analysis

The results are expressed as the mean ± SD (unless otherwise noted). A two-way ANOVA was performed for each dependent variable, assuming sex and training as fixed factors and the rats as a random factor (SAS**®** 9.2, Cary, NC, USA). Whenever a significant F-value was obtained, a *post-hoc* test with a Tukeýs adjustment was performed for multiple comparison purposes. The associations between the mechanical tests, histomorphometric parameters, COL I protein expression, and qPCR grouped by gender were estimated using Pearson or Spearman correlations, depending on the distribution of the variables. The significance level was set at p<0.05.

## Results

The body weight analysis revealed that the male rats were significantly heavier than the females, irrespective of training (main effect of sex; p<0.0001). Similarly, both the weight and length of the femur were greater in the male rats when compared with the females, irrespective of training (main effect of sex; p<0.0001 and p<0.0001, respectively) (Table 2).

**Table 2:** General characteristics of the animals.

	FEMALE		MALE	
Parameter	Sedentary (n = 10)	Trained (n = 11)	Sedentary (n = 10)	Trained (n = 9)
Initial body weight (g)*	224.9±9.9	224.5±10.2	325.9±19.4	323.3±35.1
Final body weight (g)*	252.9±17.2	241.7±10.6	448.8±35.7	441.4±32.4
Left femur body (g/100 g of body weight)*	0.75±0.05	0.78±0.06	1.1±0.09	1.1±0.6
Length of the left femur (cm)*	3.3±0.1	3.3±0.1	4.5±0.4	4.4±0.2
Initial maximum speed (m/min)	23.0±4.9	28.0±2.0	22.5±4.9	27.0±2.7
Final maximum speed (m/min)	27.0±5.8	49.0±4.9#	28.0±3.1	51.5±3.9#

* indicates main effect of sex (p<0.0001);

# indicates p<0.05 for within-sex comparisons with both the initial values and with the final speed of the sedentary animals.

The maximum speed achieved by the animals was similar between the sexes at the beginning of the study. The male and female trained rats significantly improved their maximum speed during the test when compared with both their initial values (p<0.0001) and with their sedentary counterpart values at the end of the study (p<0.0001), demonstrating the efficacy of the training program in improving the exercise capacity (Table 2).

Table 3 describes the results of the mechanical parameters of the femur. The maximal force and stiffness were significantly lower in the female rats than in the males, irrespective of training (main effect of sex; p<0.0001 and p<0.0001, respectively). The resilience was positively affected by exercise in the males (p = 0.04; within-sex comparison) but not in the females (p = 0.89; within-sex comparison). Conversely, the exercise did not affect the fracture load in any of the groups (female: p = 0.83; male: p = 0.89; within-sex comparisons). However, the males presented a higher fracture load than the females, irrespective of training (main effect of sex; p<0.0001).

**Table 3:** Mechanical property parameters of the right femur of the sedentary and trained animals.

	FEMALE		MALE	
Parameter	Sedentary (n = 10)	Trained (n = 11)	Sedentary (n = 10)	Trained (n = 9)
Maximal force (N)*	101.5±10.5	100.9±14.1	134.5±21.2	146.9±23.2
Resilience (10^−3^ Joule)* ^ #^	0.036±0.01	0.042±0.01	0.039±0.02	0.062±0.02
Stiffness (10^3 ^N/m)*	247.5±34.9	249.5±35.3	318.9±26.0	344.5±36.5
Fracture load (k/N)*	79.2±36.5	89.9±20.5	114.3±31.6	105.0±28.9

* indicates main effect of sex (p<0.05) ^#^ indicates main effect of training (p = 0.01).

**Figure 1:**
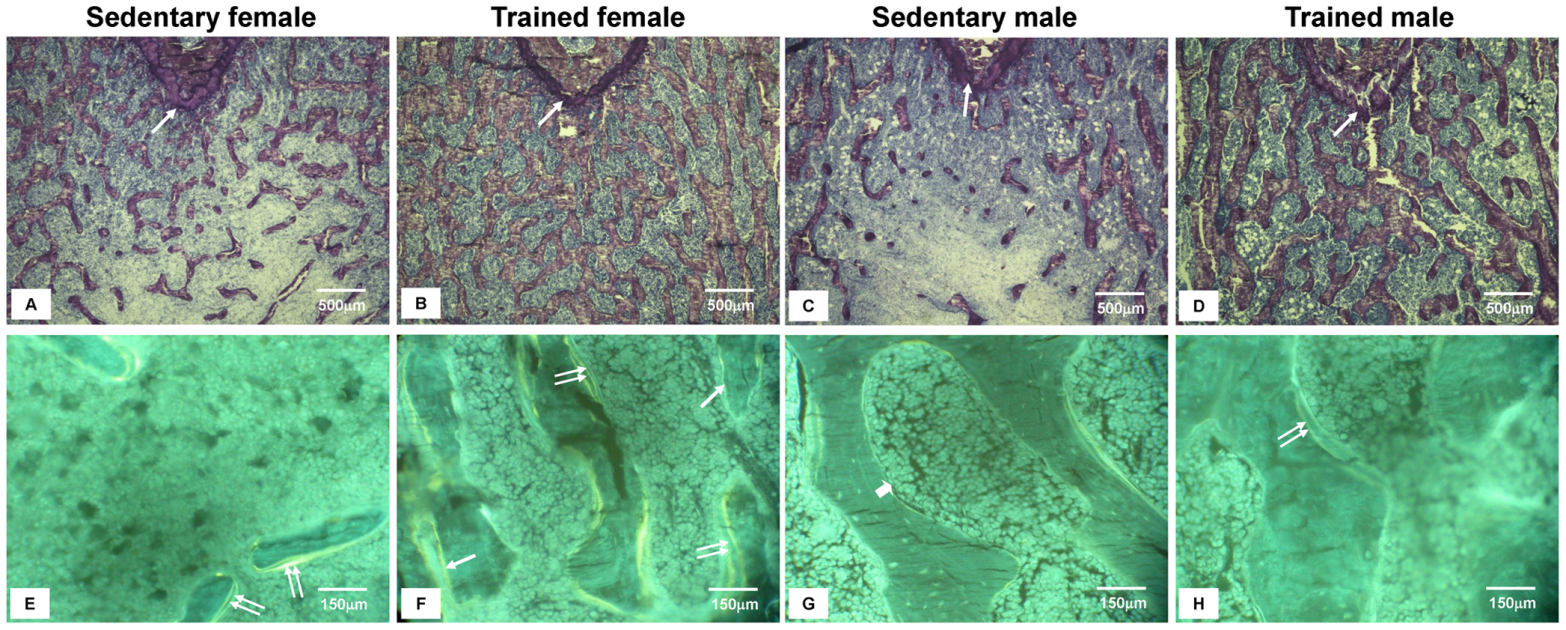
Illustrative bone histological characteristics of sedentary and trained animals. 1A–1D. Undecalcified Bone: Characteristic light microscopy aspects of trabecular bone (femoral metaphysis). Toluidine blue staining showing an increase in the trabecular bone volume (BV/TV) and trabecular thickness (Tb.Th) in the trained animals (B and D) compared with their sedentary counterparts (A and C). The epiphyseal growth plate is indicated by arrows. Histomorphometric analyses were performed at 195 µm under the epiphyseal growth plate (Magnification, x40). 1E–1H. Double oxytetracycline labeling: Characteristic fluorescent light microscopy of undecalcified bone (femoral metaphysis). Unstained bone sections under UV light of the sedentary (E and G) and trained (F and H) animals. Single and double labels are indicated by the single and double arrows, respectively. By quantifying the distance between the oxytetracycline double-labels, we observed that the trained males (H) presented a greater mineral apposition rate (MAR) than the sedentary males (G) and trained females (F). By evaluating the percentage of the trabecular bone surface that was double-labeled, we calculated the bone formation rate (BFR/BS), which was increased only in the trained males (H) (Magnification, x250). Details of the histomorphometric results can be found in Table 4.

With respect to the structural parameters of the histomorphometric analysis, BV/TV was significantly greater in the females than in the males, irrespective of training (p<0.001; main effect of sex). Importantly, the exercise training was effective in changing this parameter in both sexes (female: p = 0.005; male: p = 0.005; within-sex comparisons) (Figures 1A–1D). The Tb.N was significantly higher in the females when compared with males, and the exercise training did not affect this parameter (main effect of sex; p<0.0001). Similarly, the Tb.Sp was not affected by training, but a main effect of sex was observed (p<0.0001), with the males presenting higher values than the females. In contrast, Tb.Th was positively changed by exercise in the males (p = 0.0001; within-sex comparison) but not in the females (p = 0.1; within-sex comparison). N.Ot/B.Ar was positively affected by training only in the male rats (p = 0.002 within-sex comparison). The analysis of the formation parameters revealed that the male rats presented a higher MAR compared with the females (main effect of sex; p = 0.0004). Exercise training was effective in changing not only the MAR but also the BFR/BS in the males (p = 0.01 and p = 0.03, respectively; within-sex comparisons) but not in the females (p = 0.86 and p = 0.91, respectively; within-sex comparisons) (Figures 1E–1H). Finally, the analysis of the resorption parameters showed that a main effect of sex was found for Oc.S/BS, and ES/BS (p<0.0001 and p<0.0001, respectively), with the males presenting higher values for both variables than the females, regardless of training (Table 4).

**Table 4:** Histomorphometric analysis of the trabecular bone parameters in the femur.

	FEMALE		MALE	
	Sedentary (n = 10)	Trained (n = 11)	Sedentary (n = 10)	Trained (n = 9)
**Structural parameter**				
Trabecular Volume (BV/TV, %)*	37.1±3.6	43.5±5.3#	30.0±3.0	36.0±4.0#
Trabecular Number (Tb.N,/mm)*	6.1±0.3	6.3±0.5	5.1±0.5	4.8±0.7
Trabecular Thickness (Tb.Th, µm)	61.8±8.0	69.0±5.1#	59.0±6.2	76.0±11.0#
Trabecular Separation (Tb.Sp, µm)*	103.0±5.0	91.0±16.0	141.0±21.0	135.0±23.0
Number of Osteocytes (N.Ot/B.Ar,/mm^2^)	407.2±116.7	389.2±52.4	486.4±79	552.6±133#
**Formation parameter**				
Osteoid Thickness (O.Th, µm)	1.2±0.1	1.4±0.2	1.2±0.1	1.3±0.2
Osteoid surface (OS/BS, %)	4.8±3.0	6.0±3.4	5.0±2.2	5.6±1.4
Osteoblast surface (Ob.S/BS, %)	3.6±2.4	4.7±2.7	3.9±2.5	4.7±1.1
Osteoblast number (N.Ob/T.Ar,/mm^2^)	21.0±9.9	25.0±14.0	16.0±9.2	20.0±8.5
Mineralizing surface (MS/BS, %)	3.7±2.0	4.5±1.5	3.7±1.4	4.5±0.9
Mineral apposition rate (MAR, µm/day)*	0.53±0.2	0.59±0.1	0.64±0.1	0.88±0.1#
Bone formation rate (BFR/BS, µm^3^/µm^2^/day)	0.02±0.02	0.02±0.01	0.02±0.005	0.04±0.008#
**Resorption parameter**				
Eroded Surface (ES/BS, %)*	4.4±1.96	3.4±1.6	6.3±3.0	6.5±1.8
Osteoclast Surface (Oc.S/BS, %)*	0.93±0.41	0.85±0.53	1.56±1.0	1.57±0.46
Osteoclast number (N.Oc/T.Ar,/mm^2^)	3.62±1.5	3.31±2.1	4.37±2.2	4.32±1.4

* indicates main effect of sex (p<0.05);

# indicates p<0.05 for within-sex comparisons (sedentary *vs.* trained animals).

**Figure 2:**
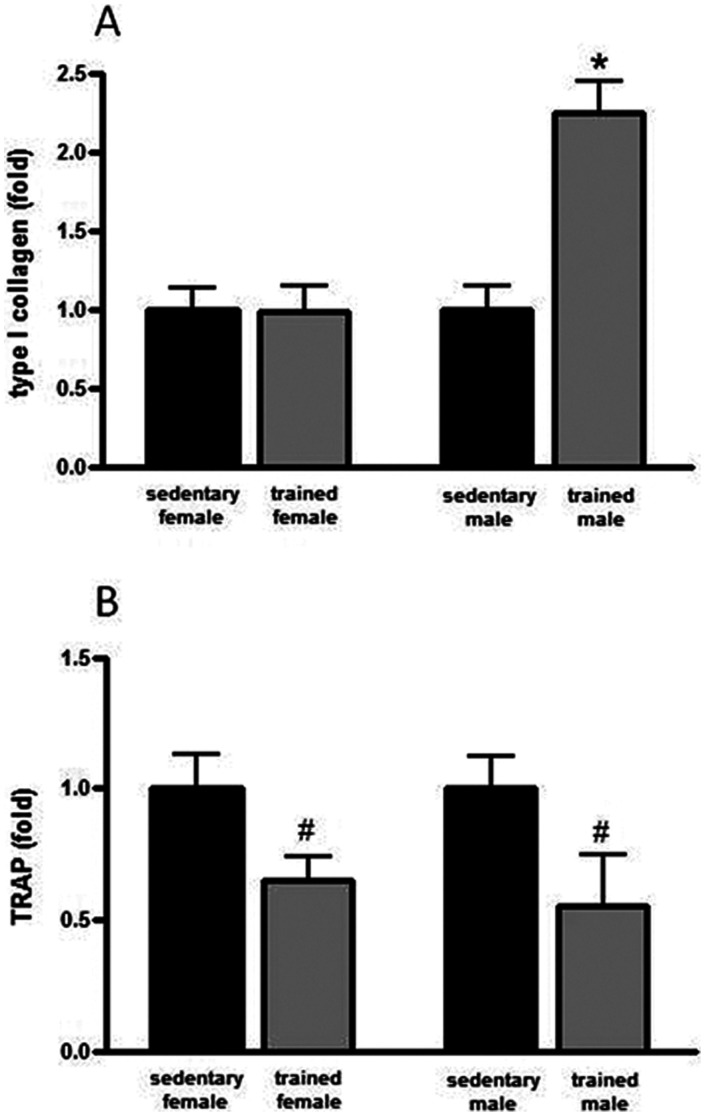
mRNA expression of COL I (panel A) and TRAP (panel B) (data are expressed as the mean and SEM). *indicates p<0.05 when compared with their sedentary counterparts (within-sex comparisons).

Corroborating the histomorphometric parameters of BV/TV, we found a significant increase in the mRNA expression of COL I (124%) in the trained male rats compared with their sedentary counterparts (p<0.05) (Figure 2A). We also observed a lower osteoclastic activity in both the female and male trained rats. The mRNA expression of TRAP decreased 35% in the trained females (p<0.05) and 45% in the trained males (p<0.05) (Figure 2B).

**Figure 3:**
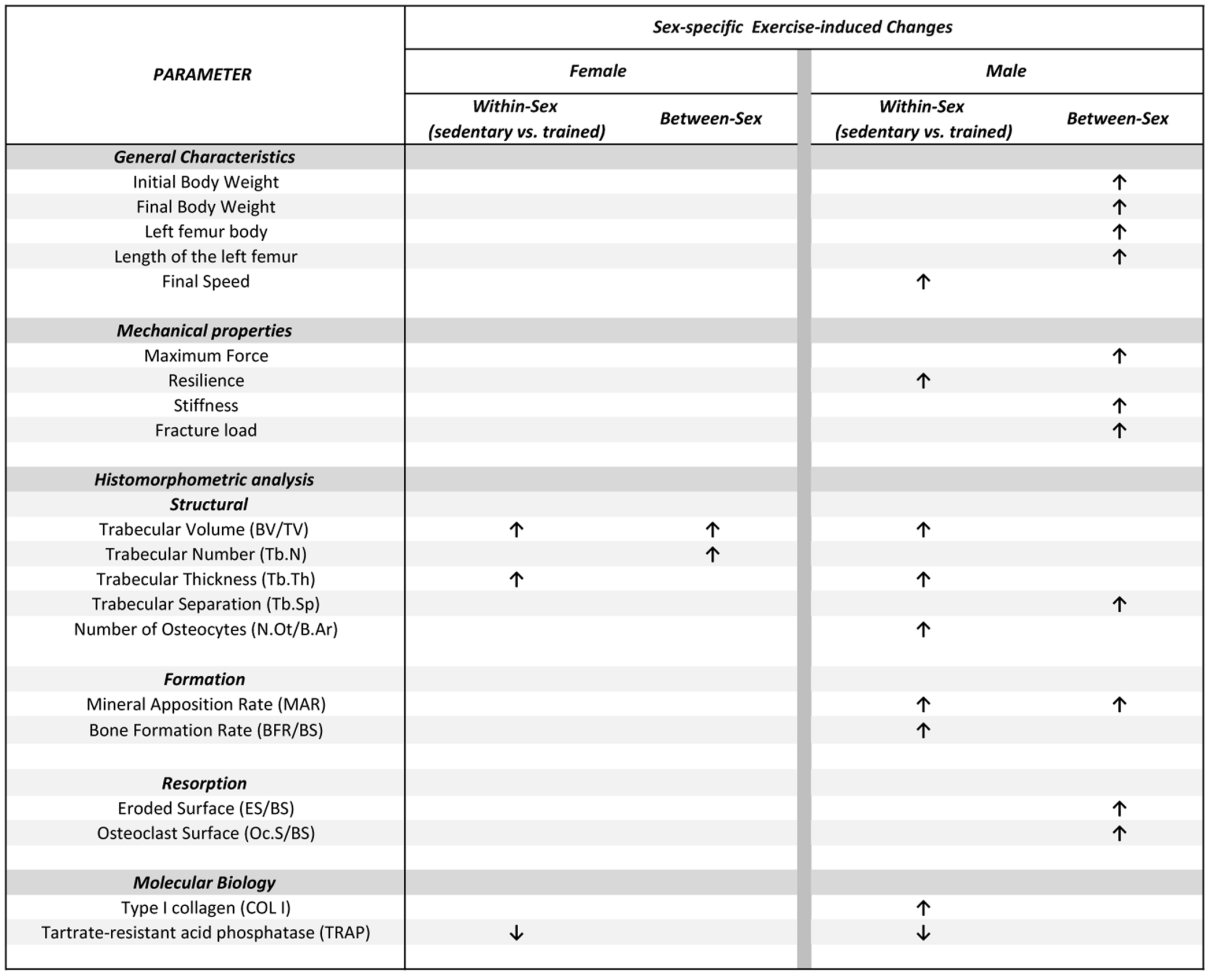
Summary of the sex-specific exercise-induced changes in bone-related general characteristics, and mechanical, histomorphometric, and molecular parameters. The “within-sex” column indicates the differences induced by exercise training either in female or male rats. The “between-sex” column indicates the differences in sex irrespective of training (i.e. main effect of sex).

The correlation analysis between the mRNA expressions of COL I and TRAP and the histomorphometric parameters revealed some significant associations. Positive associations between COL I mRNA expression and BV/TV (r = 0.77, p = 0.0147), Tb.Th (r = 0.79, p = 0.0114), and BFR/BS (r = 0.85, p = 0.0034) were found in males. Conversely, no associations were observed for any of the parameters in the female rats (p>0.05). Additionally, we observed a negative association between TRAP mRNA expression and BV/TV (r = −0.59, p = 0.0439) and a positive association with Tb.Sp (r = 0.75, p = 0.0052) and Tb.N (r = 0.66, p = 0.0207) in the female rats. The male rats displayed negative associations between the mRNA expression of TRAP and BV/TV (r = −0.65, p = 0.0159), Tb.Th (r = −0.71, p = 0.0063), MAR (r = −0.63, p = 0.0220) and BFR/BS (r = −0.71, p = 0.0063). Figure 3 depicts a summary of the sex-specific exercise-induced changes in bone-related general characteristics, and mechanical, histomorphometric, and molecular parameters.

## Discussion

Although exercise has been recognized as one of the most effective strategies to promote bone mass accrual, whether sexual dimorphism exists in response to exercise remains unknown. To gather knowledge on this topic, we investigated the effects of an exercise training protocol upon bone plasticity in young male and female rats. Collectively, our data showed that exercise yielded differential adaptations with respect to the bone structure, biomechanical properties, and molecular signaling in the bone of male and female rats.

Skeletal sexual dimorphism in bone metabolism is mainly attributed to stimulatory androgen action as opposed to inhibitory estrogen action on periosteal bone formation [8]. Importantly, skeletal growth is stimulated by mechanical loading [24], which in turn may also be influenced by sex hormones. For example, exercise may stimulate periosteal bone formation, whilst estrogen appears to have an inhibitory effect in female mice [25]. Furthermore, estrogen supplementation was able to suppress the periosteal response to mechanical loading in male rats [26]. There is evidence suggesting that estrogen and mechanical strain may also share common signaling pathways, stimulating proliferation independently in osteoblast-like cells derived from male and female rats as well as in human osteoblast cells [27]. Interestingly, estrogen receptor modulators and antagonists block the increase in proliferation in response to mechanical strain, whereas dihydrotestosterone and androgen receptor activation are apparently not involved. These findings suggest that estrogen, but not androgen receptors, influence the response to strain. Together, these findings suggest that sex hormones and their receptors may account for the difference in the mechanical response to loading between sexes observed in the current study.

Even though our findings revealed that trabecular volume was positively affected by exercise in both male and female rats (main effect of training), the trabecular thickness, resilience, mineral apposition rate, and bone formation rate increased only in the trained males, demonstrating the sexual dimorphism in response to exercise. Corroborating these observations, the number of osteocytes increased significantly only in trained males. Osteocytes are the most frequent bone cells that are able to connect to themselves and others (e.g., osteoblasts and osteoclasts) through dendritic processes, creating an extensive net that can “sense” the mechanical stimuli applied to the bone [28], [29], [30], [31]. Interestingly, mechanical loading has been demonstrated to prevent osteocyte apoptosis, possibly through the Wnt/β-catenin pathway [28]. Supporting this concept, Robling et al. [32] demonstrated that mechanical stimulation reduced the osteocyte expression of sclerostin, a negative regulator of the Wnt/β-catenin pathway, thereby increasing bone mass. You et al. [29] demonstrated that osteocytes stimulated by mechanical strain yielded alterations in the precursors of the bone marrow and in pre-osteoblasts, decreasing the formation of osteoclasts and potentially bone resorption. Therefore, one may infer that exercise-induced osteocyte activation may lead to not only increased bone formation but also decreased bone resorption. Despite the significant changes in the parameters of bone resorption (see Table 4), this speculation is only partially corroborated by our molecular findings (possibly due to large variabilities in the data), which indicated that exercise induced an increase in the COL I mRNA expression (only in males) and a decrease in the TRAP mRNA expression (in both sexes), which are genes involved in bone formation and resorption, respectively. Interestingly, in line with their attributed molecular roles, the COL I mRNA expression and TRAP mRNA expression were positively and negatively, respectively, associated with the parameters of bone remodeling (e.g., BV/TV, Tb.N, Tb.Th, MAR, and BFR/BS).

Compared with the female rats, the males had a larger body mass; bigger, thicker and heavier femurs; and greater bone maximal force and stiffness (main effect of sex). In contrast, the trabecular volume was significantly greater in females. The sexual dimorphism related to these bone morphological and biomechanical parameters may be interpreted as a differential developmental feature in our growing rats, reinforcing previous observations [8].

In conclusion, we showed that exercise may elicit differential adaptations with respect to the bone structure, biomechanical proprieties, and molecular signaling in male and female growing rats. Future experimental studies should consider the sexual dimorphism of bone in response to exercise in their study design and data interpretation.
